# Filling Proximal Cavities in Bicuspids and Molars
*The paper announced on this subject by Dr. K. B. Davis was not prepared by reason of illness.


**Published:** 1889-09

**Authors:** Edmund Noyes


ARTICLE III.

### FILLING PROXIMAL CAVITIES IN BICUSPIDS AND MOLARS.*

DISCUSSION IN ILLINOIS STATE DENTAL SOCIETY.

OPENED BY DR. EDMUND NOYES.

*Mr. President:* I must confess to less preparation than I meant to have had before addressing the Society, for I had expected to hear the paper read before discussing the subject.

There are a few points upon which I will touch briefly. These operations are among the most important and difficult to make. I think that you will agree with me that more bicuspid teeth are lost from decay than of any other class, and that this is in a very considerable degree due to the difficulty of making operations upon them that will prove permanently successful. Of course, in a brief discus-

*The paper announced on this subject by Dr. K. B. Davis was not prepared by reason of illness.

sion of this kind, I can only refer to a few of the more important aspects of the question.

One thing essential to these operations, is the contour of the teeth when they are finished. I mean by contour the shape of the crown of the tooth, including the filling, and I am sure you will bear me out in the assertion that vast multitudes of bicuspid and molar teeth are mutilated by the operations made upon them. The restoration as nearly as possible to an ideal contour is more important in respect to these teeth than to the incisors. It is important in relation to the recurrence of decay, to comfort in their use, and to the health of the gums between the teeth, thus preventing conditions favorable to the production of pyorrhœa alveolaris. In order to have perfect contour of such teeth when the operations are finished, it is indispensable that they should be separated to a considerable degree before the operation is attempted, either by slow wedging or by the positive and forcible use of the separator. Though there may appear to be sufficient space at the time of operating, unless the teeth are moved farther apart than their natural positions, the form of the teeth cannot be restored, for there will be no opportunity either to perfectly pack the outer portions of the filling or to finish it afterward. The finishing of a filling cannot be done without sufficient space for the passage of the file, tape or disc which is to do the work. If the natural contour be not restored such spaces will usually close up, the natural tendency of the arch being to fall together until all its stones, if I may so use the simile, touch and support each other. The teeth are brought more closely together at their necks than they were originally and recurrence of decay is favored at that point. For these reasons it seems to me impossible to exaggerate the importance of taking time and insisting that our patients shall submit to the wedging necessary for an abundance of room, so that at the conclusion of the work the teeth shall be further apart at their necks under the margin of the gum than they are near the grinding surface. If the teeth are nearly of the ideal form, all that need be done is to restore their original shape, but there are numerous instances in which by the removal of portions of the teeth toward their lingual surfaces, and by the careful shaping of the tooth before the filling is begun, it may approach more nearly the ideal form than nature had made it.

For my ideas on the subject of enamel cleavage as concerned in the formation of cavities, I am indebted to Dr. Black. It will not do to form the cavity in such a way as to leave enamel rods with only the outer ends remaining, and in many instances the simplest way to avoid this is to put the excavator in the cavity and split the enamel from within outward. It will break in the line of cleavage, and although it may look as if you were taking away much of the undercut, it is seldom that you break away anything that should remain. The final cutting of enamel should be to bevel the outer border, so that the gold may hang over the ends of the enamel rods. You have doubtless seen instances in which a three-cornered piece of enamel has broken away from the edge of the filling, showing that cleavage of enamel has not been considered in the preparation of the cavity. Whenever you see such a piece of enamel split out, it is evidence that there were a series of short enamel rods close to the edge of the filling which should have been removed.

The question of enamel cleavage should not be lost sight of in the preparation of cavities anywhere.

Another point of great importance is the necessity that the packing of gold should be directed toward the wall of a cavity. You have all seen operations in clinics, in which the attempt has been made to fill cavities by the application of force, which was not against nor even parallel with the anterior wall of the cavity. Gold ought to be packed toward the place where you want it to lie, and the shape of the instruments and direction of force must be consistent with this requirement.

You have observed a grinding surface filling in a molar, which was good everywhere, except on the anterior buccal corner. There was a little undercut which had not been filled, and the tooth decayed at that point. You have all seen buccal margins of fillings in bicuspids and molars, posterior fillings especially, that have failed for the same reason, because the gold was never packed perfectly against that wall. The perfection of this operation requires the most careful skill and utmost patience, and a thorough conscientiousness. Of course, the packing must be perfect to begin with, but unless the finishing is accurate, there will, in many instances, be a continued irritation of the gums between the teeth, which ought, after they recover from the punishment received in the work to be healthy and sound. It has always seemed to me indispensable for the perfection of this border that the gum should be crowded away so as to expose it fully and afford the opportunity of perfect vision and accurate workmanship at that point. I have never seen any method that would compare for effectiveness with that of driving an orange wood wedge between the teeth and crowding the gum far enough to clearly expose the cervical border of the filling. This can almost always be done, because the gum can be made to yield as far as decay has progressed in almost every instance, and a wedge holds the space which is required as well as a separator would, it reflects the light from the mirror sufficiently to show that margin clearly, and it protects the cavity from the access of mucus from the gum which may sometimes get under the edge of the rubber. It holds the teeth firmly and steadily against the concussion of the mallet, so that the painfulness of its application which is very considerable, is quite compensated for by the diminished pain of the operation, and the opportunity which it affords for perfect accuracy in the insertion and the trimming of the filling are of incalculable benefit in my judgment.

Dr. C. S. Case, Jackson, Mich.: In filling proximal cavities between bicuspids and molars, Dr. Noyes, with his usual thoroughness, has made it almost unnecessary to say more upon the subject. There were two points, however, upon which I question the propriety of proceeding as the Doctor has described, and while I do not doubt but that they work successfully with him, I believe their general adoption would be unadvisable.

The first is relative to inserting an instrument into the cavity, and breaking outward the overhanging margin of enamel along the cervical border, where the decay is quite extensive. While I believe it always desirable to remove enamel ***unsupported by dentine*** along this border, I prefer to cut it carefully away, and smooth with fine-cut burs or corundum points, rather than trust to the uncertainty of "enamel cleavage."

The second is relative to depending upon orange-wood wedges with which to obtain the proper amount of separation and perfect exposure of the cervical margin—where the decay has extended beneath the gum. A better practice, in my estimation, is in the use of a matrix—preferably of aluminium. I hardly think this material is fully appreciated for matrices. It can be easily cut, bent and pounded to any desired shape or thickness, and is of sufficient rigidity to resist all required force. For the fillings under discussion, thin and curve one edge of the matrix so that it will pass between the gum and the cervical margin of the cavity. It should fit against the root, and bent partially around the tooth. This should be held firmly in place by a wedge, but not driven in at this stage of the operation with a view of ***separating*** the teeth. If the matrix is grooved on the outside, the wedge will not be forced against the gum. Use soft gold, tin, or tin and gold, and direct the packing force toward the matrix, filling first along the margin, and building the material back into the cavity as you progress. When the cavity is partially filled, remove wedge and matrix, and finish the surface of your partial filling. Now insert a wedge, or preferably a Perry separator, resting it on one side against the filling, and separate the teeth sufficiently to fully contour the crown.

Dr. Patrick—I am not going to occupy your time by giving you instructions how to pack gold, but will simply say the more solid you pack it the better.

I desire more particularly to call your attention to one of the principal causes of failure in filling proximal cavities of bicuspid teeth. I think it is in not thoroughly understanding the exact anatomy of the part concerned in the operation.

I will draw as well as I can a transverse section, at the neck, of a bicuspid tooth. I will endeavor to show you as clearly as possible why it is difficult to fill a cavity in that situation. Notice the shape of the tooth at this point. There is a depression on either side which gradually increases toward the apex of the tooth, forming a septum between the two canals. Generally there is a slight blending between these canals, giving something like a dumb-bell form. I care not what kind of matrix you use, you never can adapt it to this depression. You will have a shoulder there which it is impossible to get rid of unless you use the little saws of our worthy president, or one of the draw-files.

Dr. Crouse—I apprehend that the most difficult thing is finishing the filling after it is made. It is not an easy matter to pack gold properly at this point; it can be done better by other force than the mallet—a force by which you can use the opposing teeth as a fulcrum, and get heavy pressure by a turn of the instrument.

I have very little use for matrices. I prefer in such cavities to start the filling with a slender border of non-cohesive gold. One of the essential elements in these cases is plenty of room, but how are you going to get it? We must use one or both forms of separator, the Parr or Perry, and I prefer the Parr, because if you want to use force quickly, you get it much more easily by this instrument.

Another point, and an important one in my opinion, is the white, chalky condition of the teeth. You have undoubtedly noticed a little streak of white pearl or decalcified enamel running beyond a proximal cavity in the form of a T on each side. What would you do with it? I have been in the habit of burring out and carefully filling that portion of the cavity first, then I have, as it were, a plain cavity to finish.

The sandpaper discs of various shapes have been to me invaluable. I am a strong advocate of contouring proximal surfaces where the patients take proper care of them.

There is another difficulty which occurs with proximal cavities. You will frequently find extensive caries under the enamel cusps. You will have large undercuts, and such undercuts are difficult to fill with gold. It is good practice to fill these spaces first with oxychloride or oxyphosphate of zinc, and I prefer the former, making the cavity more simple for the gold.

Dr. Johnson—When Dr. Crouse alluded to the use of the matrix, I knew very well he would condemn it. I have used it in certain cases with success, and I spoke in favor of it a year ago.

At the twenty-fifth anniversary meeting of the Chicago Dental Society, I had the honor of giving a clinic. I filled a distal cavity in a bicuspid, using a matrix. Dr. Crouse was one of the Executive Committee, and when the cavity was half filled—just about the time when criticism should be made—I called him up and said, "I want you to criticise that filling." He looked at it and said, "I have no criticism to make on it," and now he comes here with the same old story and condemns the use of the matrix.

Dr. Crouse—I do not use it because I can get along better without it.

Dr. Johnson—I have such confidence in your operative ability that, if there is any man in this society that can use a matrix, you can if you wish.

There is another point: I understood you to say that you would leave an overhanging wall of enamel here at this point and fill with cement. It seems to me we should not leave the wall overhanging to the extent indicated by this diagram.

I agree with what has been said about sandpaper discs. I do not think they are used as much as they ought to be.

Another point that has not been mentioned, is the trimming of the margins of cavities just before introducing the filling. Some one, I think, has alluded to trimmiug these margins with burs. You can make a better margin by the expert manipulation of sandpaper discs than with a bur, and the work can be done in half the time. If a filling overlaps the border at the top, I cut away the buccal and lingual corners; I can trim it in a few moments with a sandpaper disc, leaving a nice surface and margin.

Dr. Case mentioned the use of small corundum points for this purpose. The S. S. White Dental Manufacturing Company have made small diamond and copper points of different shapes, and I apprehend that these are going to be useful appliances for beveling margins. Perfumed vaseline has been recommended to make discs pliable, and I find it useful.

Dr. Patrick—The last speaker is so enthusiastic in regard to the use of the matrix that he is under the impression that I have abandoned it, but I wish to relieve his mind, for I use it frequently. I use it for all it is worth, and not a particle more.

Dr. Tibbetts—Failure after the operation, is due to some pathological or traumatic cause. To obviate this possibility prepare the cavity with a view to anticipating such a result. I think one of the greatest sources of failure has not been mentioned, namely, the point of contact with the contiguous teeth. Decay does not occur in the first instance at the point of contact, but immediately below; where particles of food and secretions lodge and decompose. It is always better to cut below the line of retention of food, because secondary decay is prevented. To avoid the possibility of failure from the retention of food, it is essential to cut away enough of the proximal surfaces to prevent recurrence of decay when the teeth are in position.

A traumatic source of failure may occur in a very perfect filling by the surfaces of the teeth rubbing together in the process of mastication. Some of the most beautiful operations that are made, where an overhanging edge of enamel has been left, will soon return with the corners of the enamel broken off, necessitating a secondary operation. A full and free cutting of the enamel walls almost parallel, facilitates the operation of packing the gold.

The speaker then illustrated on the board his method of preparing and filling a cavity as described.

Dr. Sitherwood—I wish to endorse what Dr. Crouse has said. I suppose every gentleman present knows how to prepare a proximal cavity in bicuspid teeth. But why must we use matrices? I had the misfortune—or, perhaps, good fortune—in my early life to learn a trade that required more manual dexterity than the filling of any proximal cavity. Why use a matrix when you can fill a cavity without it just as well? For separating I use a little cotton saturated with melted wax, leaving it twenty-four hours. This will separate the teeth sufficiently. We may, if we wish, use a Parr or Perry separator. It requires manual dexterity to fill teeth properly with any form of gold, no matter what kind of instrument is used, whether hand plugger, automatic plugger, or a mallet. We should not confine ourselves to one method or instrument in packing gold, but use whatever seems best adapted to the case.

I do not care to use matrices, for I can fill teeth more quickly without them.

Dr. Brophy—Some of the remarks made this evening I heartily indorse, others I wish to criticise.

With reference to matrices, there is not an instrument made by any man that will serve our purpose in all cases. Each instrument we have we use for a certain purpose. We cannot use one plugger to fill all cavities. Statements have been made to the effect that it is essential to have a matrix adapt itself to the proximal surface of the cavity closely. This is what a matrix should never do. I hope I shall hear that statement no more. If it is properly adjusted it never adapts itself to the proximal surfaces involved. If it should be so adapted, even approximately, the result will be a failure. The dentist who seeks to force a matrix to the border, and hold it there, certainly has not learned how to use matrices. I think one of the most common causes of failure in filling proximal cavities in bicuspids and molars is in the preparation of the cavity itself. In preparing a cavity we often leave sharp angles. We may polish them off a little with sand-paper, but we are apt to leave them. When we begin to insert gold these angles are pulverized under the force of the plugger. The filling may look fairly well when finished, but disintegration follows at these points.

Dr. Morrison, of St. Louis—To my mind the popular cause of failure is that when a dentist discharges his patients he fails to give them the proper advice as to the care of these surfaces. We all have doubtless observed that where patients care for these surfaces they do not decay. There is no doubt that fillings are made every day that should save these teeth, if dentists would do their duty by insisting upon proper care.

Dr. Koch—I have a criticism to make on the remarks of the last speaker. He says the cause of failure is, that dentists do not give their patients proper advice as to the cleanliness of their teeth. Now, if this has anything to do with failure at all, I believe it is because patients *do not follow* the advice of their dentists.

Dr. Noyes—It is my belief that the question of cutting out the grinding surface should depend upon the thickness of the overhanging enamel and in relation to the extent or depth of decay in the cavity. We ought to separate the teeth enough so that we can operate upon them. With reference to the opportunity to do the work, of course, if there is no separation we are compelled to cut from the grinding surfaces in order to make the operation. I think we ought to have plenty of room. We should consider the overhanging enamel in relation to the depth of decay.

The extension of cavities is most necessary, not at the beginning of the surface intrinsically, but toward the lingual and buccal walls in the direction of the natural extension of decay, and there is where the failure to make sufficient cutting often takes place.

There is one point more I want to touch upon, and that is in regard to the contour of teeth. The extension of a cavity must be as far as disintegration of the enamel has taken place in almost every instance, but occasionally we will see a space or section or two at this point (illustrating) where decay takes place. It is sometimes possible to increase the convexity of the surface at the expense of a portion of this enamel. I do not think the enamel should be cut through to expose the dentine in ordinary cases of this kind, but the tooth may be made more convex by taking off some of the thickness of the enamel on either side, so that the convexity at the point of greatest danger is greatly increased and the disintegration is removed, so that the filling need not be carried quite so far toward the buccal and lingual faces as would otherwise be necessary. It must be admitted that in a good many of the cavities filled of this kind decay will recur. It depends more upon patients paying attention to our instructions than any other one thing.

Dr. Case—I hope the gentlemen present did not understand from my remarks that I always use a matrix for proximal fillings. I would like to say to Dr. Sitherwood that for fifteen years I filled teeth without using it. For the last five years I have used it considerably, but with caution, and believe that I fully appreciate the fact that the matrix is a dangerous adjunct in slovenly hands, but nevertheless a valuable one when properly managed.

I would say to Dr. Patrick that I had no idea or expectation of giving instruction to this body of *how* to pack gold. My object being to show how, by directing the force at first, as discribed, the material could be forced between the smoothed and beveled cervical margin, and the matrix; crowding the latter outward sufficient to fill the cavity a little more than full, for finishing purposes—alternate pieces being wedged back into the cavity to secure a firm and solid position to the whole.

